# Post-Neoadjuvant Treatment Strategies for Patients with Early Breast Cancer

**DOI:** 10.3390/cancers14215467

**Published:** 2022-11-07

**Authors:** Elisa Agostinetto, Flavia Jacobs, Véronique Debien, Alex De Caluwé, Catalin-Florin Pop, Xavier Catteau, Philippe Aftimos, Evandro de Azambuja, Laurence Buisseret

**Affiliations:** 1 Institut Jules Bordet, L’Université Libre de Bruxelles (U.L.B.),1070 Bruxelles, Belgium; 2 Curepath Laboratory (CHU Tivoli, CHIREC), Rue de Borfilet 12A, 6040 Jumet, Belgium; 3 Department of Pathology, Erasme Hospital, Université Libre de Bruxelles, route de Lennik 808, 1070 Brussels, Belgium

**Keywords:** breast cancer, pathological complete response, post-neoadjuvant treatment, residual disease

## Abstract

**Simple Summary:**

Treatment strategies for early breast cancer have significantly improved in the last decades. Several new effective agents have proved clinical benefit and have entered the clinics, changing the treatment landscape for this disease and inducing significant prolongation of patient survival. Alongside, there has been an evolution in the design of clinical trials for early breast cancer, with an increasing interest in the pre-surgical treatment approach, which allows a direct evaluation of treatment effect on tumor size and a post-therapy risk stratification. Consequently, the post-neoadjuvant setting has been gaining increasing attention, thanks to the possibility to provide additional treatment for selected patients at higher risk of relapse, namely those who did not respond to neoadjuvant therapy and had residual disease at surgery.

**Abstract:**

Pre-surgical treatments in patients with early breast cancer allows a direct estimation of treatment efficacy, by comparing the tumor and the treatment. Patients who achieve a pathological complete response at surgery have a better prognosis, with lower risk of disease recurrence and death. Hence, clinical research efforts have been focusing on high-risk patients with residual disease at surgery, who may be “salvaged” through additional treatments administered in the post-neoadjuvant setting. In the present review, we aim to illustrate the development and advantages of the post-neoadjuvant setting, and to discuss the available strategies for patients with early breast cancer, either approved or under investigation. This review was written after literature search on main scientific databases (e.g., PubMed) and conference proceedings from major oncology conferences up to 1 August 2022. T-DM1 and capecitabine are currently approved as post-neoadjuvant treatments for patients with HER2-positive and triple-negative breast cancer, respectively, with residual disease at surgery. More recently, other treatment strategies have been approved for patients with high-risk early breast cancer, including the immune checkpoint inhibitor pembrolizumab, the PARP inhibitor olaparib and the CDK 4/6 inhibitor abemaciclib. Novel agents and treatment combinations are currently under investigation as promising post-neoadjuvant treatment strategies.

## 1. Introduction

Breast cancer (BC) is the most common cancer in women worldwide, with more than 450,000 new cases every year in Europe [1,2]. Most patients with BC (around 65%) are diagnosed with early-stage disease, a condition that is potentially curable with standard locoregional and systemic treatments. However, up to 30% of them experience disease recurrence after surgery, either with local or distant metastases [3].

Neoadjuvant chemotherapy (NAC), originally used for the treatment of unresectable, locally advanced, or inflammatory BC, has become increasingly adopted in earlier stages and represents now the standard of care for many forms of early BC. Neoadjuvant treatments offer several advantages over adjuvant ones. First, it may downstage the disease providing a better surgical outcome with less extensive breast and axillary surgery. Second, it can be used to test in vivo the activity of new agents and therapeutic strategies by monitoring tumor size during the treatment. Additionally, it may allow escalation or de-escalation of further systemic treatment according to the response to neoadjuvant treatment.

In the large meta-analysis (*n* = 11,955) of the Collaborative Trials in Neoadjuvant Breast Cancer (CTNeoBC) led by Cortazar and colleagues, patients with early BC who achieved a pathological complete response (pCR) after NAC had better long-term survival than patients with residual disease, and this association was even more pronounced in patients with more aggressive subtypes, namely triple-negative BC (TNBC) and HER2-positive (HER2+), hormone receptor-negative tumours [4].

**Figure 1:**
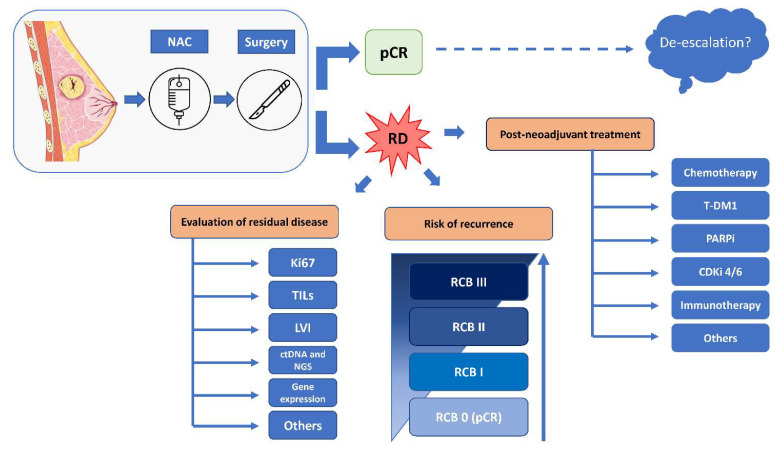
Simplified representation of post-neoadjuvant treatment strategy for breast cancer patients. Patients eligible for preoperative treatment receive neoadjuvant chemotherapy. If at the time of surgery the tumor is no longer detectable in the specimen (i.e., a pathological complete response (pCR) is achieved), these patients could be potentially candidate to de-escalation treatment strategies, as pCR is associated with lower risk of disease recurrence. The potential de-escalation strategy can apply to surgery, radiotherapy, or systemic adjuvant therapy. Of note, the management of a patient with a pCR is multifactorial, and requires multidisciplinary discussion. Indeed, caution should be paid to avoid the removal of too many treatment components. In case of invasive residual disease, patients may be considered for additional post-neoadjuvant treatments, for instance with chemotherapy (i.e., capecitabine in triple-negative breast cancer) or antibody drug conjugates (i.e., T-DM1 in HER2-positive breast cancer). The evaluation of residual disease can be done using various biomarkers for risk assessment (e.g., Ki67, TILs, RCB, gene expression, and genetic alterations). Residual disease can be classified according to the Residual Cancer Burden index, that combines pathologic measurements of primary tumor (size and cellularity) and nodal metastases (number and size), and classifies the specimen in one of four classes (RCB 0, i.e., pCR, RCB I, RCB II, RCB III). A higher RCB index (i.e., RCB III) indicates a larger amount of residual disease, and it is associated with a higher risk of recurrence.Abbreviations: CDKi 4/6: cyclin-dependent kinase inhibitor; ctDNA: circulating tumor DNA; HER2, human epidermal growth factor receptor 2; HR+, hormone receptor-positive breast cancer; LVI: lymphovascular invasion; NAC: neoadjuvant chemotherapy; NGS: next-generation sequencing; PARPi: poly ADP-ribose polymerase inhibitor; pCR: pathologic complete response; RCB: residual cancer burden; RD: residual disease; TDM1: trastuzumab-emtansine; TILS: tumors-infiltrating lymphocytes.

Patients who achieve pCR are at lower risk of disease relapse and may therefore be candidates for de-escalation treatment strategies, conversely to patients with residual disease at surgery who are at higher risk for disease recurrence and could benefit from additional post-NAC treatments (Figure 1).

To date, some systemic post-NAC therapies have already been approved in clinical practice for patients with residual disease at surgery (e.g., capecitabine for patients with TNBC and T-DM1 for patients with HER2-positive disease), and many other novel strategies are under investigation. The aim of this review is to examine the currently available post neoadjuvant treatment strategies and to explore the more promising treatment strategies that are being investigated in clinical trials.

## 2. Triple-Negative Breast Cancer

**Figure 2:**
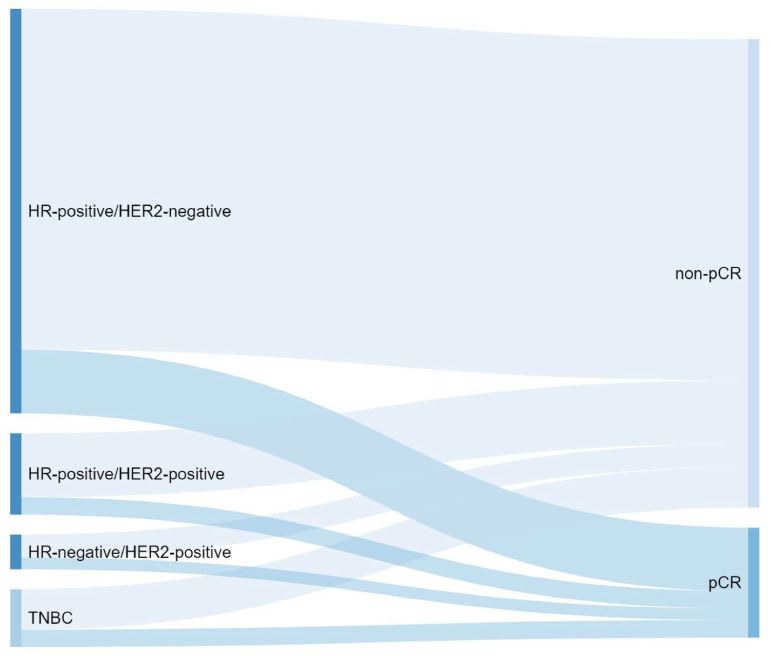
Proportion of pathological complete response (pCR) after neoadjuvant therapy according to breast cancer subtypes. HR-positive/HER2-negative (HR+/HER2−) is the most prevalent subtype in breast cancer, occurring in approximately 70% of patients, followed by HER2−positive (HER2+) and TNBC subtypes (approximately 20% and 10%, respectively). Subtype-specific pCR rate are 8.3% in HR+/HER2−, 18.7% in HER2+/HR+, 38.9% in HER2+/HR− and 31.1% in TNBC (original figure based on literature data, i.e., Houssami et al., Meta-analysis of the association of breast cancer subtype and pathologic complete response to neoadjuvant chemotherapy, Eur J Cancer 2012. doi: 10.1016/j.ejca.2012.05.023) [9]. Abbreviations: HR: hormonal receptors; TNBC: triple-negative breast cancer; pCR: pathological complete response.

TNBC is an aggressive BC subtype with limited treatment options [5]. Neoadjuvant polychemotherapy regimens remain the standard of care for early-stage TNBC larger than 2 cm or with positive nodes [6]. Only rare histological subtypes, such as secretory or adenoid cystic carcinomas that are at low risk of recurrence, or very early-stage tumors (T1aN0) may avoid chemotherapy [6]. The rate of pCR after NAC in TNBC has consistently improved in the last decades, thanks to the introduction of new effective treatment strategies. The addition of carboplatin to NAC regimen has demonstrated to significantly improve the pCR rate in patients with TNBC (pCR rate: 37% with chemotherapy regimen not containing carboplatin vs. 52% with chemotherapy regimen containing carboplatin [7]). Moreover, the addition of pembrolizumab to NAC has further increased this rate up to 65% [8]. However, despite the improvements in treatment strategy, a relevant proportion of patients with TNBC still do not achieve a pCR after completion of NAC (Figure 2), and are at higher risk of disease relapse. To improve the outcomes of this high-risk population, several agents have been tested in the post-neoadjuvant setting in recent years [4].

The CREATE-X trial (Table 1) was the first phase III study to show the benefit of additional capecitabine after completion of NAC. In this trial, 910 patients with HER2-negative disease and residual tumor after NAC with anthracyclines and taxanes were randomly assigned to either observation or six to eight cycles of adjuvant capecitabine. Both the endpoints of disease-free survival (DSF) and overall survival (OS) were significantly better in the experimental group, especially in the TNBC subgroup (N = 286, hazard ratio [HR] 0.58, 95% confidence interval [CI] 0.39–0.87 for DFS and HR 0.52, 95% CI 0.30–0.90 for OS) [5]. Post-neoadjuvant capecitabine now represents the standard of care for patients with TNBC and residual disease after completing NAC [6,10,11].

Among TNBC, patients harboring germline BRCA1/2 mutations represent a specific high-risk subgroup. The OlympiA trial (Table 1) evaluated the addition of one year of treatment with oral olaparib for patients with high-risk HER2-negative early BC and a germline BRCA mutation, either in the adjuvant or post-neoadjuvant setting [13]. In the second prespecified event-driven analysis of OS, survival rates at 4 years were 89.8% with olaparib vs. 86.4% with placebo, yielding a 3.4% absolute improvement (HR = 0.68, 98.5% CI 0.47–0.97; *p* = 0.009). The updated invasive DFS (iDFS) and distant DFS (DDFS) were consistent with previous results (HR, 0.63; 95% CI, 0.50–0.78 and HR, 0.61; 95% CI, 0.48–0.77 respectively), both favoring treatment with olaparib over placebo [14].

While the benefit of this post-NAC was consistent for patients with germline mutations, the role of somatic mutations in the residual tumor after NAC remains undefined so far. In the BRE12-158 trial, residual tumors after NAC were sequenced with a next-generation sequencing assay and patients were randomly assigned to four cycles of genomically targeted therapy versus treatment of physician’s choice (TPC). This study failed to demonstrate the superiority of personalised therapy over standard therapy [24].

A subsequent phase III trial by the ECOG-ACRIN group failed to demonstrate the superiority of post-neoadjuvant treatment with carboplatin or cisplatin over capecitabine in TNBC patients with residual disease after NAC [17].

Immunotherapy has revolutionized the treatment of several cancer types, including BC. Several clinical trials in early TNBC [8,16,25] have already shown improvement in pCR rate when immunotherapy is added to standard chemotherapy, and are detailed below.

The KEYNOTE-522 trial (Table 1) evaluated the addition of pembrolizumab to NAC with carboplatin and paclitaxel followed by doxorubicin-cyclophosphamide in patients with stage II-III TNBC. Patients receiving pembrolizumab had a higher pCR rate (64.8% vs. 51.2%, 95% CI 5.4–21.8, *p* < 0.001), which was the primary endpoint of the study [8]. Moreover, the estimated event-free survival (EFS) at 36 months was 84.5% in the experimental group, as compared with 76.8% in the standard group (HR 0.63, 95% 95% CI 0.48–0.82, *p* < 0.001) with an absolute 7.7% improvement [15]. Based on this result, FDA approved pembrolizumab in combination with NAC followed by pembrolizumab as a single agent in the adjuvant treatment of high-risk early-stage TNBC [8]. Of note, this is the only study that provided a platinum agent in the chemotherapy backbone.

The IMpassion031 trial (Table 1) was also conducted in stage II-III TNBC, but patients received atezolizumab as immune checkpoint inhibitor (ICI) and the backbone chemotherapy used in the neoadjuvant phase was nab-paclitaxel without carboplatin. Atezolizumab was continued for 1 year after surgery. The addition of atezolizumab resulted in a statistically significant increase in pCR rate (57.6% vs. 41.1%, 95% CI 5.9–27.1, *p* = 0.0044) [16]. IMpassion031 was not powered for the evaluation of EFS, DFS and OS, and these results are still immature (medians not reached for any of the above-mentioned endpoints).

The Gepar-Nuevo trial tested in the neoadjuvant setting a different ICI, durvalumab, in combination with nab-paclitaxel followed by standard anthracycline-based chemotherapy, in patients with TNBC. Although the trial did not meet its primary endpoint of improving pCR, the addition of durvalumab to NAC improved 3-year iDFS from 76.9% to 84.9% and OS from 83.2% to 95.1%. Interestingly, ICI was not continued in the adjuvant phase. Of note, the study was not powered to detect a survival difference, and these results need further confirmation [26].

Although PD-L1 expression is an established predictive biomarker in the advanced setting, it does not discriminate between responders and no responders in the early setting. Indeed, both patients with PD-L1 positive and negative tumors derived a benefit from the addition of ICIs. The benefit of immunotherapy in early disease seems to be independent of PD-L1 status, although PD-L1-positive tumors are more likely to respond [27].

Although there is still a need to better identify patients who will benefit from neoadjuvant ICIs, these results leave three major questions that need to be answered by future studies in the post-neoadjuvant setting: (i) What is the best backbone chemotherapy? (ii) How can capecitabine and olaparib be integrated with ICIs in case of invasive residual disease after NAC? (iii) Is it possible to de-escalate ICI treatment once pCR has been achieved [28]?

The first question is of particular relevance, as the NAC regimen could influence the activity of the ICI, and, ultimately, their clinical benefit. In the phase II TONIC trial [29], patients with advanced TNBC were randomized to receive two weeks of induction therapy with either cyclophosphamide, doxorubicin, cisplatin or irradiation, followed by nivolumab. Patients who received doxorubicin and cisplatin as induction showed the highest overall response rates (35 and 23%, respectively). Consistently, in the NeoTrip trial, testing the addition of atezolizumab to an anthracycline-free chemotherapy regimen in the neoadjuvant phase, no increase in pCR was observed. Although these effects might be subtle and hard to assess from the indirect comparison of clinical trials, these data suggest that the choice of chemotherapy backbone could have a major impact on ICI efficacy. Extremely large studies should be designed to provide a final answer to this question. Thus far, after the approval of pembrolizumab based on KEYNOTE-522 data, an anthracycline-based chemotherapy regimen including carboplatin represents the preferred chemotherapy regimen in this setting.

For the second question, so far no data are available in the early setting. In clinical practice, adjuvant treatment of patients with residual disease can be tailored according to residual cancer burden (RCB), overall recurrence risk, and BRCA germline status. In patients at high risk of recurrence, it may be appropriate to provide adjuvant treatment with capecitabine in case of residual disease and to continue treatment with pembrolizumab if started in NAC. In BRCA-mutated patients, olaparib is recommended for 1 year if there is a high risk of recurrence. The question is whether a PARP inhibitor should be added to pembrolizumab when used in the neoadjuvant phase. The potential side effects of this combination need to be carefully weighed, as no robust data are available in the early setting yet. Conversely, in patients with low RCB and low overall risk of recurrence, the continuation of treatment with pembrolizumab alone could be a reasonable option if no immune-related toxicities occurred during the neoadjuvant phase.

Finally, the KEYNOTE-522 trial showed that the addition of pembrolizunab for patients who achieved pCR was associated with only 2% improvement in 3-years EFS, whereas a 10% of difference was observed in patients who did not achieved pCR. These results raised the question of whether de-escalation of adjuvant treatment might be an option for patients achieving pCR with chemo-immunotherapy. However, in the absence of prospective evidence, pembrolizumab in adjuvant treatment should also be considered as the standard therapy in patients with pCR.

Several new approaches for post-neoadjuvant treatment of TNBC are ongoing (Table 2). The phase III study SASCIA is comparing the antibody drug conjugate (ADC) sacituzumab govitecan to TPC as post-neoadjuvant treatment of HER2-negative BC patients with residual disease after NAC (NCT04595565). The phase II study ASPRIA is evaluating the combination of sacituzumab govitecan and the anti-PD-L1 atezolizumab, in patients with residual disease and, additionally, with detection of circulating tumor DNA (ctDNA) after the completion of NAC with or without an ICI (NCT04434040). The phase III study ZEST will test post-neoadjuvant niraparib in patients with TNBC or HER2-negative, BRCA mutated breast cancer with ctDNA detection after surgery or adjuvant therapy (NCT04915755). The phase II study COGNITION-GUIDE is a seven-arm umbrella trial aiming to evaluate genomics-guided post-neoadjuvant therapies in patients with early breast cancer (NCT05332561).

## 3. HER2-Positive Breast Cancer

Neoadjuvant therapy represents the standard of care for most HER2+ early BC (i.e., tumor size > 2 cm or positive lymph nodes) [6]. Patients with residual disease at surgery are at increased risk of recurrence, and post-neoadjuvant treatment strategies have been investigated to improve their long-term outcomes [30,31].

Currently, the results of two large studies [22,23] in this setting have shown the efficacy of a new treatment strategy, which has led to a change in the current treatment indications (Table 1).

In the landmark phase III KATHERINE trial, 1486 patients with residual disease after NAC with anti-HER2 therapy were randomized to post-neoadjuvant T-DM1 or continuation of trastuzumab for 14 cycles. Regarding the NAC regimen, 77.9% of patients in the T-DM1 arm and 75.9% in the trastuzumab arm received an anthracycline-based chemotherapy, respectively. In addition, 18.7% of patients in the T-DM1 group received dual anti-HER2 blockade with trastuzumab and pertuzumab in the neoadjuvant phase, compared to 17.9% in the control arm. Of note, 68% of enrolled patients in both groups had lymph node involvement at diagnosis. After a median follow-up of 41.4 months, 3-year iDFS was 88.3% in the T-DM1 group vs. 77.0% in the trastuzumab arm (HR 0.50, 95% CI, 0.39–0.64, *p* < 0.001). Distant recurrence occurred in only 10.5% of patients treated with T-DM1 compared with 15.0% of patients treated with adjuvant trastuzumab. Based on these significant results, T-DM1 was approved in 2019 for the post-neoadjuvant treatment of patients with HER2+ early BC with residual disease after NAC [22].

The second major study was the ExteNET trial, which tested one year of treatment with oral neratinib, an irreversible pan-HER tyrosine kinase inhibitor (TKI), versus placebo in 2840 patients with HER2+ disease after completion of standard chemotherapy (NAC or adjuvant) and one year of adjuvant trastuzumab. Patients were stratified by hormone receptor status, and those with hormone receptor positive tumors (25% in the experimental arm and 27% in the placebo arm) also received adjuvant endocrine therapy (ET), according to local guidelines. At the primary analysis at 2 years, neratinib was associated with a significant improvement in the primary endpoint of iDFS in patients with hormone receptor positive tumors, with an HR of 0.67 (*p* = 0.008), and this benefit was confirmed in the final 5-year analysis (HR 0.73, *p* = 0.008) [24]. Based on these results, in June 2018, the EMA approved adjuvant neratinib in combination with ET for high-risk patients with hormone receptor positive, HER2 + BC.

Interestingly, the subgroup of patients with residual invasive disease after NAC showed clinically meaningful improvements in DFS and OS, but these results should be interpreted with caution due to the exploratory nature of the analysis and the small number of patients [32].

Several other treatment strategies are currently under investigation, with ADCs, TKIs, ICIs, and vaccines [33] being tested in the post-neoadjuvant phase in HER2+ BC patients (Table 2). In particular, the promising results of ADCs in advanced setting justify their use at an earlier stage [34,35]. Notably, DESTINY-Breast05 (NCT04622319), a large phase III trial, is testing the efficacy of trastuzumab-deruxtecan (T-DXd) or T-DM1 in patients with HER2+ early BC without pCR after NAC. Two phase III studies, CompassHER2-RD trial (NCT04457596) and Astefania (NCT04873362), are evaluating the addition of tucatinib or atezolizumab, respectively, to standard T-DM1 in patients with residual disease after completion of NAC.

Finally, other de-escalating strategies are currently under evaluation. In the recently published PHERGAIN study [36], early metabolic response by FDG-PET showed promise to identify patients who will benefit from anti-HER2 therapy and, therefore, can be spared of additional chemotherapy. The ongoing phase II DESCRESCENDO trial (NCT04675827) is investigating the administration of subcutaneous pertuzumab and trastuzumab in patients with hormone receptor negative, HER2+ tumors who have achieved pCR after NAC with an anthracycline-free regimen (i.e., paclitaxel, pertuzumab, and trastuzumab).

## 4. Hormone Receptor-Positive Breast Cancer

The magnitude of response to NAC varies significantly according to BC subtype, and, hormone receptor positive, HER2-negative BC are less likely to respond to neoadjuvant chemotherapy than other biologic subtypes (Figure 2) [4]. Therefore, several attempts have been made to find effective post-NAC strategies in this subset of patients, not only based on pCR (less suitable in this BC subtype), but also considering other scores (e.g., CPS-EG) [37].

Data showing that the addition of CDK4/6 inhibitors (CDK4/6i) to ET improves survival in patients with advanced hormone receptor positive tumors provided the basis for investigating these agents in the early setting [38]. To date, the only trial specifically investigating the benefit of adding CDK4/6i to patients with residual disease after NAC is the PENELOPE-B trial, a phase II study in which 1250 patients with residual disease after taxane-containing NAC and at high risk of relapse (CPS-EG score ≥ 3 or ≥2 with ypN+) were randomized to receive 13 cycles of palbociclib 125 mg daily or placebo added to ET. After a median follow-up of 42.8 months, palbociclib failed to improve iDFS compared to placebo (HR 0.93, 95% CI 0.74–1.17) (Table 1) [18]. The PALLAS study was a phase III trial evaluating the addition of palbociclib to adjuvant ET in patients with stage II or III hormone receptor positive BC. Patients were eligible to participate regardless of any response to NAC, and 33.7% of the patients (n = 1939) had previously received NAC. At the final analysis, palbociclib failed to improve iDFS (iDFS at 4 years: 84.2% vs. 84.5%; HR 0.96; 95% CI, 0.81–1.14; *p* = 0.65), and the trial was closed early for futility [17,39]. Another trial testing CDK4/6i in the adjuvant setting is the MonarchE trial, which evaluated the addition of 2 years of adjuvant abemacicilb to standard ET in hormone receptor positive, HER2-negative early BC patients who were at high risk of relapse after surgery [20]. In this study, 37% of patients had previously received NAC. The high-risk population was defined as: (i) ≥4 positive nodes; (ii) 1–3 positive nodes and at least one of the following: tumor size ≥5 cm, histologic grade 3, or central Ki-67 ≥ 20%. After a median follow-up of 27 months, the addition of abemaciclib to standard adjuvant ET significantly improved 3-year iDFS from 83.4% to 88.8% (HR 0.70, 95% CI 0.59–0.82; *p* < 0.0001) and DDFS from 86.1% to 90.3% (HR 0.69, 95% CI 0.57–0.83; *p* < 0.0001). Interestingly, subgroup analysis indicated that the iDFS benefit of adjuvant abemaciclib was more pronounced in the subgroup of patients treated with NAC (N = 2087, HR 0.63, 95% CI 0.50–0.80) than in patients treated with adjuvant chemotherapy (N = 3289, HR 0.75, 95% CI 0.58–0.97) [40]. Based on these results, abemaciclib received FDA approval in 2021 as adjuvant therapy for women with high-risk, node-positive, hormone receptor positive, HER2-negative BC with Ki-67 ≥ 20% with one of the following features: (i) N1 disease and other high-risk features (T3, high grade) or (ii) N2 or N3 disease.

Other agents have been investigated as treatment options for patients with high-risk hormone receptor positive BC. In the afore-mentioned CREATE-X trial, 601 patients had hormone receptor positive disease with residual invasive disease after NAC with anthracycline, taxane, or both. In this subgroup of patients, adjuvant capecitabine did not improve DFS (76.4% vs. 73.4%, HR 0.81; 95% CI, 0.55–1.17) nor OS (93.4% vs. 90.0%, HR 0.73; 95% CI, 0.38–1.40) [12]. In the Olympia trial, 18.2% of patients receiving adjuvant olaparib had hormone receptor positive, HER2-negative breast cancer at high risk of relapse, defined as patients with residual disease after NAC and a CPS + EG score of 3 or more (in the neoadjuvant group) or 4 positive lymph nodes or more (in the adjuvant group) [13]. In the subgroup analysis, despite the relatively low number of patients, the iDFS benefit of olaparib appeared to be less pronounced in patients with hormone receptor positive disease (*n* = 325) (neoadjuvant group: HR 0.52, 95% CI 0.25–1.04, and adjuvant group: HR 1.36, 95% CI 0.41–4.71). Nevertheless, it should be acknowledged this was a small subset of patients, and hormone receptor positive tumors tend to recur later, thus longer follow-up is required.

For neoadjuvant treatment of high-risk hormone receptor positive, HER2-negative BC, there are two phase III trials currently ongoing: the KEYNOTE-756 (NCT03725059), in which pembrolizumab is used in both the neoadjuvant and adjuvant phases, and CheckMate 7FL (NCT04109066), in which nivolumab is used in both settings as well.

## 5. Post-Neoadjuvant Locoregional Treatment

The optimal locoregional treatment after NAC depends on response to NAC and therefore this response should be carefully followed with regular physical examinations and imaging techniques (i.e., magnetic resonance imaging (MRI), mammography, ultrasound, and, if indicated, PET/CT). Currently, there is no consensus on the best imaging method for assessing response in patients receiving neoadjuvant therapy. Guidelines recommend that patients receiving neoadjuvant therapy (either chemotherapy or endocrine therapy) should be monitored for response with clinical breast examination at regular intervals and breast imaging may be used to confirm clinical suspicion of progression and for surgical panning [41]. When imaging is used, the recommendation is that the most informative modality at baseline—either mammography, ultrasound or MRI—should be used at follow up [41]. However, level of evidence is considered insufficient and the strength of the recommendation moderate [41], highlighting the need of more studies assessing this question [42]. Recently, an expert consensus highlighted the importance of MRI when evaluating tumor response in order to guide the surgical management of early BC after NAC [42].

Data from literature [43] suggest that MRI and ultrasound have a superior performance compared to mammography and clinical breast examination in patients treated with NAC, particularly when residual tumor is present. In this regard, although mammography and ultrasound are common methods used to establish tumour size at the time of diagnosis, they may underestimate tumour size. Although surgical planning is mainly driven by the residual tumour after response to neoadjuvant therapy, misleading tumour size at baseline may also be important, as an underestimation may directly lead to surgery patients who would benefit from a neoadjuvant approach.

Furthermore, even with a complete clinical response, the presence of residual invasive disease cannot be excluded because of the modest correlation between tumor measurements by physical examination, imaging, and tumor size at final pathologic analysis [44,45,46].

Regarding the surgery of the primary breast tumor, either by breast conserving surgery (BCS) or by mastectomy, the standard treatment is to operate within the residual tumor margins after NAC. An EBCTCG meta-analysis [47] including more than 4700 patients from 10 clinical trials on NAC, showed that NAC was associated with an increased rate of local recurrences [42]. Although caution is required to interpret these data (as some systemic and surgical treatments were outdated (dating back to 1983), as well as the postoperative imaging, and some patients where surgery was omitted were included in the analysis), these findings warn about the risks of an excessive treatment de-escalation, that should be avoided.

After NAC, axillary management consider the nodal status at diagnosis and after the treatment. More specifically, if the patient has a clinically negative axilla (cN0) before treatment, sentinel lymph node biopsy (SLNB) is sufficient. A meta-analysis of 16 studies on 1456 BC women with initial cN0 who received NAC, supports the safety and feasibility of SLNB over axillary lymph node dissection (ALND) for axillary staging in these patients [48].

On the other hand, if the patient already has axillary lymph node involvement (confirmed radiologically or pathologically) prior to NAC, treatment of the axilla consists of either SLNB, targeted axillary dissection (TAD), ALND, and/or axillary radiation. The choice among these procedures depends on the extent of lymph node involvement prior to NAC and the response to treatment. If in the past, ALND was the only treatment option for patients with a nodal involvement at diagnosis, the approach has now changed with the introduction of NAC, as it can be possible to de-escalate surgical treatment in selected patients, such as those who show a complete response after NAC. Nonetheless, in case of extensive nodal involvement at diagnosis (cN2 or cN3), ALND followed by locoregional nodal irradiation should be performed, regardless of clinical response to NAC. For patients with minor nodal involvement (cN1) prior to NAC, treatment depends on the response to NAC: patients with persistent clinically positive axilla (ycN1) after NAC should undergo ALND followed by locoregional nodal irradiation. In contrast, patients who are clinically node-negative (ycN0) after NAC can be considered for SLNB or TAD, with selective removal of the node that was initially positive by biopsy [49]. In this case, if SLNB or TAD is negative at final pathology (ypN0), no ALND is required.

NAC rises a challenge to adjuvant locoregional RT indications, as these were traditionally exclusively based on the pathology report of the primary surgery. Both baseline and post-NAC surgery tumor characteristics and stage can affect the risk of disease recurrence in patients treated with NAC, hence both should be considered to define the best locoregional treatment approach. Most guidelines recommend locoregional RT for all patients with stage III disease at diagnosis, regardless of response to NAC [6,11,50]. On the other hand, patients with cT1-2 N0 breast cancer who respond to NAC do not benefit from locoregional RT [6,11,50]. RT indications for patients with cT1-2 N1 BC are more debated.

In a prospective registry study (RAPCHEM, BOOG 2010–03) [51] including 838 patients with cT1–2N1 BC treated with NAC and surgery, patients were randomized to one of three RT strategies (whole breast radiation therapy (WBRT) or post-mastectomy radiation therapy (PMRT) +/− axilla level I-II +/− axilla level III-IV) according to their risk category (low, intermediate, high, respectively). The 5-year rate of loco regional recurrence was 2.2%, overall, and, specifically, 2.1%, 2.2% and 2.3% in the low, intermediate, and high-risk group, respectively. These findings suggest that de-escalation of RT according to risk in selected patients with cT1–2N1 breast cancer treated with NAC and surgery is possible and safe from an oncological point of view [51].

Ongoing randomized trials (such as NRG Oncology/NSABP B-51/RTOG 1304) will provide additional useful information in the RT management of patients with pathologically positive axillary nodes at diagnosis (cT1-2 cN1) and who are ypN0 after NAC (NCT01872975)

In patients who present a positive sentinel node after NAC (ypN1sn), a randomized trial (Alliance A11202) investigates the use of ALND + locoregional RT (without axillary RT) versus locoregional + axillary RT (without ALND) (NCT01901094). Hence, the Alliance A11202 trial is similar to the EORTC AMAROS trial (axillary RT compared to ALND in pN1(sn) post-operative setting without NAC) [52], but transposed to the post-NAC ypN1(sn) setting. Analogously to AMAROS, the Alliance trial hypothesizes that axillary RT can replace ALND with less lymphoedema and non-inferior locoregional control.

Finally, when NAC is indicated, optimizing and coordinating the timing of the different treatment modalities (i.e., systemic therapy, surgery, RT) is important [53]. Capecitabine, olaparib and abemaciclib are generally started after RT completion in order to avoid potential toxicity (concomitant use is under debate and investigation), whereas trastuzumab, pertuzumab and T-DM1 can safely be given concomitantly with RT [54].

## 6. Pathological Evaluation of Residual Disease

As the response to NAC is crucial to determine the adjuvant treatment modalities, it is important to standardize the evaluation of tumor response at surgery [55]. The pathological evaluation of post-NAC samples remains the gold standard for assessing tumor response to neoadjuvant treatments. It is extremely important to determine the degree of pathological response, which may differ between the primary tumor and the axillary lymph node metastases, and to evaluate the histological and biological characteristics of the residual tumor in both the breast and the axilla [56,57]. Pathologists have adopted a different approach for the macroscopic assessment, extent of sampling, and microscopic analyses of post-NAC surgical samples. Indeed, NAC can induce numerous tissue changes and even the identification of the primary tumor bed can be challenging because it resembles to fibrotic breast tissue. It is therefore crucial to localize the tumor before starting neoadjuvant treatment (e.g., by clips) [58]. In their evaluation, pathologists report the size of the primary tumor bed in three dimensions and the size and number of any residual neoplastic foci, along with the distance of the tumor bed/residual tumor from the surgical margins in the case of breast-conserving surgery [55,59]. In case of pathological partial response (pPR), the appearance of the residual tumor may be nodular, sclerotic, or can appear as multipli foci in the surrounding area that may be edematous and/or sclerotic. The number of tissue blocks to be collected vary according the extent of the surgical specimen [60].

In case of pCR, the tumor bed may present as an area of vascularized hyalinization, with foamy macrophages, lymphocytes, multinucleated giant cells, and hemosiderin-laden macrophages, in absence of normal ductal and lobular structures at microscopic examination [46]. In residual tumors, cells have a cellular and nuclear pleomorphism, multinucleation, an increase or decrease in cell size, bizarre giant cell forms, or an increase or decrease in nuclear-to-cytoplasmic ratio. The tumor cell cytoplasm may appear hypereosinophilic or vacuolated [59]. Most commonly, post-NAC tumors present with a lower grade due to a decrease in mitotic activity, but a subset may demonstrate a higher grade due to increased nuclear pleomorphism [59]. Recent publications have demonstrated that post-NAC grade and proliferation activity after therapy evaluated by the mitotic rate component of the Nottingham histologic grade are prognostic and should be reported [61].

As mentioned above, evaluation of response to NAC in the lymph nodes is of paramount importance, for possible surgical treatment de-escalation [53]. All axillary lymph nodes from patients treated with NAC should be sectioned at ≤2 mm intervals and those without evidence of residual tumor should be evaluated in their entirety [56,59]. In some patients, there is no sign of response in lymph nodes that are histologically identical to lymph nodes from patients who have not undergone NAC. Histologic changes related to treatment effect include lymphocyte depletion and stromal fibrosis or hyalinization which foamy macrophages and/or hemosiderin-laden macrophages [55,59]. Number of lymph nodes examined including those with residual disease or with fibrosis in the absence of residual disease as well as the extent of any residual disease (e.g., macrometastases, micrometastases, isolated tumor cells) and the presence of extracapsular extension are considered for the pathologic staging according to the latest TNM edition [55].

In addition to the ypTNM for pathologic quantification of residual disease after NAC, the AJCC recommends to evaluate the Residual Cancer Burden (RCB) that refers to the degree of residual disease after NAC [61]. RCB is associated with survival outcomes, especially in more aggressive BC subtypes, namely TNBC and HER2+ [62]. RCB is the most used grading scores in the United States and in many other countries [58]. RCB assessments depend on tumor bed size, tumor cellularity, number of positive lymph nodes, and size of the largest lymph node metastasis. For both pPR and pCR, the pathology report should be adequate and include information on histologic subtype, size, number of residual neoplastic foci, fibrosis, lymphovascular invasion, neoplastic emboli, presence of any intraductal component, and status of the margin, if applicable.

Currently, there is no consensus guidelines for the re-testing of biomarkers after NAC, and practices vary widely [54]. The College of American Pathologists recommends that if biomarkers were negative prior to therapy, re-testing should be performed on residual invasive carcinoma after NAC. An algorithmic approach might be useful to standardize the identification of cases where re-testing is appropriate [61].

## 7. Conclusions

As illustrated in our review, the collaboration among pathologists, oncologists, surgeons, and radiotherapists is essential to ensure the best management of patients with BC treated with NAC. The post-neoadjuvant setting is gaining increasing importance in BC care, thanks to a better patient selection that allows tailoring treatments according to the risk of recurrence. Response to NAC can guide the escalation or de-escalation of treatment strategies and post-neoadjuvant strategies have already demonstrate to improve survival in case of residual disease in all BC subtypes. Hence, the postneoadjuvant setting has been gaining a relevant interest in the last years, and an increasing development of clinical trials in this setting is expected in the next future.

## Figures and Tables

**Table 1:** Main studies on the post-neoadjuvant setting in breast cancer.

	Study	Population (N )	Intervention	Comparator	Results
HER2-negative/TNBC	CREATE-X [12]	HER2-negative with residual disease after NAC (910)	Capecitabine	No adjuvant therapy	5y DFS: 74.1% vs. 67.6%, HR 0.70 5y OS: 89.2% vs. 83.6%, HR 0.59
		TNBC (286)			5y DFS: 69.8% vs. 56.1%, HR 0.58 5y OS: 78.8% vs. 70.3%, HR 0.52
	OlympiA [13,14]	Overall (1836) TNBC (1509)	Olaparib	Placebo	4y iDFS: 82.7% vs. 75.4%, HR 0.63 3y DDFS: 86.5% vs. 79.1%, HR 0.61 4y OS: 89.8% vs. 86.4%, HR 0.68
	KEYNOTE-522 [8,15]	1174	NAC + pembrolizumab and adjuvant pembrolizumab	NAC and adjuvant placebo	pCR: 64.8% vs. 51.2%3y EFS: 84.5% vs. 76.8%, HR 0.63
	Impassion-031 [16]	455	NAC + atezolizumab	NAC	pCR: 58% vs. 41%
	ECOG-ACRIN EA1131 [17]	410	Platinum	Capecitabine	3y iDFS: 42.8% vs. 53.5%, HR 1.16
Hormone receptor positive	PENELOPE-B [18]	1250	Palbociclib	Placebo	3y iDFS: 81.2% vs. 77.7%, HR 0.93
	PALLAS [19]	5796	Palbociclib + ET	ET	4y iDFS: 84.2% vs. 84.5%, HR 0.96
	MonarchE [20,21]	Overall (5637)	Abemaciclib + ET	ET	3y iDFS: 88.8% vs. 83.4%, HR 0.69 3y DRFS: 90.3% vs. 86.1%, HR 0.68
		Prior NAC (2087)			3y iDFS: HR 0.69
	OlympiA [12]	Hormone receptor positive (325)	Olaparib	Placebo	3y iDFS: 83.5% vs. 77.2%, HR 0.70
HER2 positive	KATHERINE [22]	1486	T-DM1	Trastuzumab	3y iDFS: 88.3% vs. 77.0%, HR 0.50
	ExteNET [23]	Overall (2840) Hormone receptor positive ≤ 1y trastuzumab (1334)	Neratinib	Placebo	5y iDFS: 90.8% vs. 85.7%, HR 0.58 8y OS: 91.5% vs. 89.4%, HR 0.79

Abbreviations: DDFS: distant disease-free survival; DRFS: distant relapse-free survival; EFS: event-free survival; ET: endocrine therapy; HR: hazard ratio; HER2: human epidermal growth factor receptor 2; iDFS: invasive disease-free survival; NAC neoadjuvant chemotherapy; OS: overall survival; pCR: pathological complete response; T-DM1: trastuzumab emtansine; TNBC: triple-negative breast cancer.

**Table 2:** Ongoing phase II/III trials in the post-neoadjuvant setting in breast cancer.

Trial	Design	Status	Population	Treatment	Endpoint
HER2-negative/TNBC					
SASCIA NCT04595565	III	Recruiting	RD after NAC	Arm A: Sacituzumab govitecan Arm B: TPC (capecitabine or platinum-based)	iDFS
SWOG S1418/BR006 NCT02954874	III	Active, not recruiting	High-risk after NAC	Arm A: Observation Arm B: Pembrolizumab	iDFS
A-Brave NCT02926196	III	Active, not recruiting	High risk after NAC	Arm A: Avelumab Arm B: Observation	DFS
ASPRIA trial NCT04434040	II	Recruiting	RD and ctDNA after NAC	Sacituzumab + atezolizumab	Rate of undetectable ctDNA-6 Cycles
BreastImmune03 NCT03818685	II	Active, not recruiting	RD after NAC	Arm A: RT + Nivolumab and Ipilimumab Arm B: RT + Capecitabine	DFS
PERSEVERE NCT04849364	II	Recruiting	RD after NAC based on plasma ctDNA positivity and genomic marker	ctDNA positive with a genomic target: Arm 1a: DNA Repair pathway (talazoparib + capecitabine) Arm 1b: Immunotherapy pathway (atezolizumab + capecitabine) Arm 1c: PI3K Pathway (inavolisib + capecitabine) Arm 1d: DNA Repair + Immunotherapy (talazoparib + atezolizumab + capecitabine) ctDNA positive without a genomic target: Arm 2: Capecitabine or TPC ctDNA negative: Arm 3: observation, capecitabine or TPC	2y DFS
PHOENIX DDR/Anti-PD-L1 Trial NCT03740893	IIa	Recruiting	RD after NAC	Arm A: Standard of care Arm B: AZD6738 (selective ATR kinase inhibitor) Arm C: Olaparib Arm D: Durvalumab	Change in mean proliferation index (Ki67)
NCT03872388	II	Recruiting	RD after NAC	Arm A: Atorvastatin +/− capecitabine Arm B: Observation +/− capecitabine	Proportion of patients with undetectable CTCs at 6 months
NCT04197687	II	Recruiting	RD after NAC	Arm A: T-DM1 + TPIV100 and Sargramostim Arm B: T-DM1 + Placebo	iDFS
NCT04437160	II	Recruiting	RD after NAC	Arm A: Epirubicin or Pirarubicin Arm B: Cyclophosphamide	RFS
NCT02445391	II	Recruiting	RD after NAC	Arm A: Platinum based CT Arm B: Capecitabine	iDFS
APOLLO NCT04501523	II	Recruiting	High risk identified with ctDNA after NAC	Arm A: ctDNA positive, non-pCR: Tislelizumab (anti-PD1) + capecitabine Arm B: ctDNA positive, non-pCR: capecitabine Arm C: ctDNA positive, pCR: capecitabine Arm D: Follow up	5y iDFS
OXEL NCT03487666	II	Active, not recruiting	RD after NAC	Arm A: Nivolumab Arm B: Capecitabine Arm C: Nivolumab + capecitabine	PIS at week 6
NCT04677816	II	Recruiting	Vitamin D deficient in pre and post neoadjuvant setting	Arm A: Vitamin D Supplementation Arm B: Observation	pCR in vit D group
ZEST NCT04915755	III	Recruiting	BRCA wild type with ctDNA after definitive therapy	Arm A: Niraparib Arm B: placebo	DFS
COGNITION-GUIDE NCT05332561	II	Not yet recruiting	High-risk patients with RD after NAC	Genomics-guided targeted therapy (including ICI, PARPi, ADC, PI3Ki, AKTi, anti-HER2 therapy)	iDFS
MK-3475-522/KEYNOTE-522 NCT03036488	III	Active, not recruiting	High-risk early stage pre and post neoadjuvant setting	Neoadjuvant → Adjuvant Arm A: CT + Pembrolizumab → Pembrolizumab Arm A: CT + Placebo → Placebo	pCR EFS
HER2-positive					
DESTINY-Breast05 NCT04622319	III	Recruiting	RD after NAC	Arm A: T-DXd Arm B: T-DM1	iDFS
DESTINY-Breast11 NCT05113251	III	Recruiting	High-risk early stage pre and post neoadjuvant setting	A: neoadjuvant T-Dxd B: T-Dxd followed by taxane + trastuzumab pertuzumab C: AC followed by taxane + trastuzumab pertuzumab	pCR
CompassHER2-RD NCT04457596	III	Recruiting	High risk patients with RD after NAC	Arm A: T-DM1 + Tucatinib Arm B: T-DM1 + Placebo	iDFS
CompassHER2-pCR NCT04266249	II	Recruiting	pCR after NAC with taxane + trastuzumab pertuzumab	Arm A (pCR): trastuzumab pertuzumab Arm B (no pCR): T-DM1	iDFS
Astefania NCT04873362	III	Recruiting	RD after NAC	Arm A: T-DM1 + Atezolizumab Arm B: T-DM1 + Placebo	iDFS
DECRESCENDO NCT04675827	II	Recruiting	De-escalation HER2 therapy after NAC	Neoadjuvant: taxane + pertuzumab and transtuzumab FDC SC Adjuvant: in pCR group (RCB = 0): Pertuzumab and trastuzumab FDC SC in RD (RCB = 1): T-DM1 in RD (RCB ≥2): anthracycline based CT → T-DM1	3y-RFS in HER2-enriched pCR
ATP NCT04254263	III	Recruiting	RD after NAC	Arm A: Pyrotinib 2 years Arm B: Placebo 2 years	iDFS
NCT04973319	III	Not yet recruiting	RD after NAC	Arm A: Adjuvant trastuzumab pertuzumab + Pyrotinib Arm B: Adjuvant trastuzumab pertuzumab	iDFS
PHERGAIN-2 NCT04733118	II	Recruiting	CT free pCR guided strategy	Neoadjuvant: Trastuzumab and pertuzumab FDC SC +/− ET Adjuvant: Cohort A (pCR): Trastuzumab and pertuzumab FDC SC +/− ET Cohort B: T-DM1 +/− ET 10 cycles Cohort C: T-DM1 +/− ET 10 cycles (+/− TPC before T-DM1)	3y RFI
COGNITION-GUIDE NCT05332561	II	Not yet recruiting	High-risk patients with RD after NAC	Genomics-guided targeted therapy (including ICI, PARPi, ADC, PI3Ki, AKTi, anti-HER2 therapy)	iDFS
NCT04197687	II	Recruiting	RD after NAC	Arm A no pCR: T-DM1 + TPIV100 ID and sargramostim Arm B no pCR: T-DM1 + placebo + sargmamostim pCR: trastuzumab and pertuzumab 1 year	iDFS
Hormone receptor positive, HER2-negative					
MK-3475-522/KEYNOTE-522 NCT03725059	III	Active, not recruiting	High-risk early stage pre and post neoadjuvant setting	Neoadjuvant -> Adjuvant Arm A: CT + Pembrolizumab -> ET + Pembrolizumab Arm B: CT + Placebo -> ET + Placebo	pCR EFS
CheckMate 7FL NCT04109066	III	Active, not recruiting	High-risk early stage pre and post neoadjuvant setting	Neoadjuvant -> Adjuvant Arm A: CT + Nivolumab -> ET + Nivolumab Arm B: CT + Placebo -> ET + Placebo	pCR EFS
ZEST NCT04915755	III	Recruiting	BRCA-mutated patients with ctDNA after surgery or adjuvant therapy	Arm A: Niraparib Arm B: placebo	DFS
COGNITION-GUIDE NCT05332561	II	Not yet recruiting	High-risk patients with RD after NAC	Genomics-guided targeted therapy (including ICI, PARPi, ADC, PI3Ki, AKTi, anti-HER2 therapy)	iDFS
RSBNAT NCT03638648	II	Not yet recruiting	RD after NAC	Stratified according to multiple gene test-based recurrence risk level: Cohort A: High risk: capecitabine Cohort B: Low risk: control group	2y DFS

Abbreviations: ATR: ataxia telangiectasia; CT: chemotherapy; CTC: circulating tumor cells; ctDNA: circulating tumor DNA; DFS: disease-free survival; EFS: event-free survival; ET: endocrine therapy; FDC SC: fixed-dose combination for subcutaneous injection; HER2: human epidermal growth factor receptor 2; iDFS: invasive disease-free survival; NAC: neoadjuvant chemotherapy; pCR: pathological complete response; PI3K: phosphatidylinositol-3 kinase; PIS: peripheral immunoscore; RCB: residual cancer burden; RD: residual disease; RFI: relapse-free interval; RFS: relapse-free survival; RT: radiotherapy; T-DM1: trastuzumab emtansine; T-DXd: trastuzumab deruxtecan; TNBC: triple-negative breast cancer; TPC: treatment of physician choice. “→” means “followed by”.
